# The Motor Basis for Misophonia

**DOI:** 10.1523/JNEUROSCI.0261-21.2021

**Published:** 2021-06-30

**Authors:** Sukhbinder Kumar, Pradeep Dheerendra, Mercede Erfanian, Ester Benzaquén, William Sedley, Phillip E. Gander, Meher Lad, Doris E. Bamiou, Timothy D. Griffiths

**Affiliations:** ^1^Biosciences Institute, Newcastle University, Newcastle upon Tyne, NE2 4HH, United Kingdom; ^2^UCL Institute for Environmental Design and Engineering, The Bartlett, University College London, WC1H 0NN, United Kingdom; ^3^Department of Neurosurgery, University of Iowa, Iowa City, Iowa 52242; ^4^UCL Ear Institute, London, WC1X 8EE, United Kingdom; ^5^Wellcome Centre for Human NeuroImaging, London, WC1N 3BG, United Kingdom; ^6^Biomedical Research Centre, University College London Hospitals, London, WC1E 6AB, United Kingdom; ^7^Translational and Clinical Research Institute, Newcastle University, Newcastle upon Tyne, NE2 4HH

**Keywords:** auditory, fMRI, mirror neurons, misophonia, motor system, resting state connectivity

## Abstract

Misophonia is a common disorder characterized by the experience of strong negative emotions of anger and anxiety in response to certain everyday sounds, such as those generated by other people eating, drinking, and breathing. The commonplace nature of these “trigger” sounds makes misophonia a devastating disorder for sufferers and their families. How such innocuous sounds trigger this response is unknown. Since most trigger sounds are generated by orofacial movements (e.g., chewing) in others, we hypothesized that the mirror neuron system related to orofacial movements could underlie misophonia. We analyzed resting state fMRI (rs-fMRI) connectivity (*N* = 33, 16 females) and sound-evoked fMRI responses (*N* = 42, 29 females) in misophonia sufferers and controls. We demonstrate that, compared with controls, the misophonia group show no difference in auditory cortex responses to trigger sounds, but do show: (1) stronger rs-fMRI connectivity between both auditory and visual cortex and the ventral premotor cortex responsible for orofacial movements; (2) stronger functional connectivity between the auditory cortex and orofacial motor area during sound perception in general; and (3) stronger activation of the orofacial motor area, specifically, in response to trigger sounds. Our results support a model of misophonia based on “hyper-mirroring” of the orofacial actions of others with sounds being the “medium” via which action of others is excessively mirrored. Misophonia is therefore not an abreaction to sounds, per se, but a manifestation of activity in parts of the motor system involved in producing those sounds. This new framework to understand misophonia can explain behavioral and emotional responses and has important consequences for devising effective therapies.

**SIGNIFICANCE STATEMENT** Conventionally, misophonia, literally “hatred of sounds” has been considered as a disorder of sound emotion processing, in which “simple” eating and chewing sounds produced by others cause negative emotional responses. Our data provide an alternative but complementary perspective on misophonia that emphasizes the action of the trigger-person rather than the sounds which are a byproduct of that action. Sounds, in this new perspective, are only a “medium” via which action of the triggering-person is mirrored onto the listener. This change in perspective has important consequences for devising therapies and treatment methods for misophonia. It suggests that, instead of focusing on sounds, which many existing therapies do, effective therapies should target the brain representation of movement.

## Introduction

Misophonia is a disorder of emotion processing in which ordinary day-to-day sounds, at normal volume, cause distress to the point that it has debilitating effects on the occupational, social, and domestic life of the sufferer. Typically, these sounds (termed “trigger” sounds) include eating, chewing, drinking, and breathing sounds made by people other than the sufferer. Reactions of a misophonia sufferer to trigger sounds include anger, irritation, disgust, anxiety, and, in some cases, violent rage accompanied by a strong urge to escape from the situation. Since trigger sounds are common, and almost inescapable in the company of others, misophonia can lead to social isolation, and cases of suicide and suicide attempts have been reported in the media (Nauman, 2017). Although comprehensive epidemiological data on misophonia are lacking, three studies (Wu et al., 2014; Zhou et al., 2017; Naylor et al., 2021) in undergraduate student samples found that 6%-20% had moderate to severe symptoms of misophonia.

A considerable effort has been made in the last few years to understand the brain mechanisms of misophonia. Kumar et al. (2017) showed hyperactivity of anterior insula, specifically in response to trigger sounds in the misophonia group compared with normal healthy controls. Moreover, the functional connectivity of anterior insula, in response to trigger sounds, was stronger to a number of brain regions, including core nodes of the default mode network (ventromedial prefrontal cortex and posterior cingulate cortex). Using a similar paradigm with video clips as stimuli, Schroder et al. (2019) replicated the findings of hyperactivity in anterior insula to trigger stimuli in subjects with misophonia. Although the neuroimaging studies have identified a brain network underlying misophonia, the question of why such innocuous sounds cause distress and hyperactivity of brain regions remains unanswered.

Interestingly, most of the triggers in misophonia happen to be human-generated sounds of eating and chewing (Kumar et al., 2014; Jager et al., 2020), which involve orofacial actions. Although sounds are most distressing, images and silent videos of eating and chewing can also cause distress. Trigger sounds in misophonia “automatically” elicit the emotional response (Dozier, 2015) without having any self-control, despite preserved insight into the disproportionate nature of the feelings and reactions evoked (Cavanna and Seri, 2015). Additionally, trigger sounds/actions can induce spontaneous mimicry of the triggering orofacial action in many misophonia sufferers (Edelstein et al., 2013). Since the mirror neuron system (Rizzolatti and Craighero, 2004) responds to the action of others and is known to underlie spontaneous mimicry (Prochazkova and Kret, 2017) and emotional responses (Bastiaansen et al., 2009), we speculated that a process of “mirroring” the action represented by trigger sounds might be a fundamental part of the mechanism underlying misophonia. That is, in misophonia sufferers, trigger stimuli activate the same part of motor cortex that is active during generation of trigger stimuli.

Mirror neurons are a set of neurons discovered in the motor cortex of monkeys that fire not only when the monkey performs a particular action but also when the monkey sees another individual performing the same action (Pellegrino et al., 1992; Rizzolatti and Craighero, 2004). For example, there are mouth mirror neurons related to ingestive functions, such as chewing or sucking of food and lip-smacking (Ferrari et al., 2003). Moreover, the mirror neurons can be activated not only by the sight of action but also by the sounds of the action (Kohler et al., 2002). In humans, mirroring of actions takes place in a network of brain areas, including the ventral premotor cortex referred to as the Mirror Neuron System (MNS) (Iacoboni and Dapretto, 2006). The MNS has been shown to mirror mouth (e.g., biting and chewing an apple), hand and foot actions (Buccino et al., 2001) and many others (Rizzolatti and Sinigaglia, 2010).

A defining feature of the mirror neuron system is that it associates a pattern of auditory or visual input (e.g., seeing or hearing somebody chewing) to a part of the motor cortex (orofacial motor cortex) involved in producing the motor movement associated with the input (e.g., the mouth or orofacial movements) in others. This requires connectivity between sensory and motor regions. We therefore first estimated connectivity of auditory and visual cortex to the rest of the brain in misophonia and control groups using analyses based on resting state fMRI (rsfMRI), when there was no specific stimulus or task performed. We next analyzed the change in functional connectivity of two groups in response to three categories of sounds (trigger sounds, which evoked misophonic reaction in a misophonia group; unpleasant sounds, which are perceived to be aversive by both groups and; neutral sounds). Last, activation of auditory cortex and orofacial motor cortex to the three sound categories was estimated. Put together, our data support a model of misophonia based on “hyper-mirroring” of actions of others in which there is an excessive engagement of the orofacial motor cortex by the auditory and visual sensory input associated with those actions. These results provide a new framework to understand misophonia, which has important consequences for the type of therapy and treatment options to be considered for misophonia.

## Materials and Methods

We present data from two experiments: (1) rsfMRI and (2) sound-evoked fMRI. The rsfMRI data are new. The data for the sound-evoked fMRI experiment have been published previously (Kumar et al., 2017) and are reanalyzed here.

### 

#### Subjects

Seventeen subjects with misophonia and 20 control subjects were recruited for participation in the rsfMRI study after providing written informed consent to procedures approved by the local ethics committee. The misophonia subjects were recruited via an advertisement on a misophonia support website (https://www.allergictosound.com/). Misophonia participants were first required to complete three questionnaires: (1) a misophonia questionnaire designed in our laboratory and used previously in our studies (Kumar et al., 2014, 2017); (2) the Misophonia Amsterdam Questionnaire (Schroder et al., 2013); and (3) the Misophonia Questionnaire (Wu et al., 2014). A misophonia participant was recruited for the study if all of the following applied: (1) they identified sounds of eating, breathing, or chewing as trigger sounds; (2) sounds alone could trigger the misophonic reaction (i.e., no picture or video of the person producing trigger sounds was needed along with sounds); (3) the person producing trigger sounds did not have to be a specific person, such as a close family member; and (4) they scored 10 or higher (moderate to extreme misophonia) on the Misophonia Amsterdam Questionnaire. All subjects were screened by telephone by the first and third author to confirm their symptoms and questionnaire responses, and to rule out MRI contraindications.

Controls were recruited via an advertisement on a local university website. In the advertisement, the exact purpose of the study was not mentioned. Instead, it was stated that the objective of the study was to determine brain responses to our day-to-day environmental sounds. Once participants signed up for the study, they were asked by telephone how they respond to environmental sounds, including sounds of eating and breathing. They were then asked to complete the Misophonia Questionnaire (Wu et al., 2014). If typical symptoms of misophonia were absent (e.g., responding angrily, leaving the situation in response to typical trigger sounds in misophonia), the subject was recruited. No subject who signed up for the study was incidentally identified to have misophonia. The misophonia and control groups were matched in age and sex. All participants were paid £10/h plus travel expenses.

In the sound-evoked fMRI study, 20 misophonia sufferers and 22 age- and sex-matched controls were recruited following a procedure similar to that described above (for details, see Kumar et al., 2017).

#### Experimental procedure

##### rs-fMRI

Ten minutes of rsfMRI data were acquired while participants kept their eyes open. An eye-tracker was used to check whether participants conformed to the instruction. Physiologic parameters, heart rate, and respiration were measured continuously using a pulse oximeter and respiration belt.

##### Sound-evoked fMRI

fMRI data were continually acquired during the presentation of three categories of sounds: (1) trigger sounds, which evoke a misophonic reaction in subjects with misophonia (e.g., eating/chewing sounds); (2) aversive sounds, which are perceived to be unpleasant by both groups but do not evoke a misophonic response (e.g., an infant cry); and (3) neutral sounds. A list of sounds is given in Table 1. After every sound presentation for 15 s, subjects gave two ratings: (1) how annoying the sound was (both groups) and (2) how effectively the sound triggered misophonic distress (misophonia subjects) or how antisocial the sound was, based on whether the subject would move away from the source of sound (control subjects). A total of 126 trials (42 for each sound category) were presented across 5 sessions each lasting ∼11 min. Further details of the procedure can be found from our previous publication (Kumar et al., 2017).

**Table 1.** List of sounds used in the sound-evoked experiment (Kumar et al., 2017)

Trigger sounds	Unpleasant sounds	Neutral sounds
Apple crunching	Baby crying	Busy cafe
Coughing and sniffing	Belch sound	Fan sound
Crisps eating	Buzzer sound	Faucet sound
Breathing sound	Buzzing bees	Hair dryer sound
Cutlery sounds	Dentist drill	Helicopter sound
Eating food sound 1	Female crying	Kettle boiling
Eating food sound 2	Male crying	Toilet flush
Eating salad and cutlery	Multiple dogs barking	Traffic sound
Gulping water	Vomit sound	Vacuum cleaner
Slurping	Multiple infants crying	Washing machine
Eating with slurping	Alarm sound	Rain sound
Sniffing	Toddler crying	Shower sound
Chewing	Jack hammer	Phone ringing
Packet opening and eating	Female scream	Brushing teeth

#### Functional imaging data acquisition

##### rs-fMRI

Resting state MRI data were collected on a Siemens 3 Tesla Trio whole-body MRI scanner (Siemens Healthcare) with a 32-channel head coil at the Wellcome Center for Human Neuroimaging, University College London. The subject movement was discouraged by instruction and by use of soft padding within the head coil. The acquisition parameters used were as follows: TR = 3.36 s; in-plane resolution = 3 mm isotropic; TE = 30 ms; 48 slices (covering the whole brain); matrix size = 64 × 64; echo spacing = 0.5 ms; orientation = transverse; slice tilt = −30° relative to the AC-PC line. A total of 180 volumes were acquired for the resting state.

##### Sound-evoked fMRI

All imaging data were collected on a Siemens 3 Tesla whole-body MRI scanner (Siemens Healthcare) at the Wellcome Center for Human Neuroimaging, University College London. fMRI data were acquired continuously with a 12-channel coil using a sequence that was optimized for acquisition from the amygdala and orbitofrontal cortex (Weiskopf et al., 2006).The fMRI acquisition parameters were the same as in the resting state acquisition. A total of 1005 volumes were acquired across 5 sessions. Field maps were acquired (parameters: short TE = 10 ms; long TE = 12.46 ms; polarity of phase-encoding blips = −1; EPI readout time = 37 ms) for every subject after the third session.

#### Structural data acquisition for use with rs-fMRI

A structural scan for each participant was acquired using a whole-brain quantitative multiparameter maps protocol (Weiskopf et al., 2013), with 32-channel head coil, which consisted of a total of five sequences: three FLASH sequences and two calibration sequences for correcting field inhomogeneities (Lutti et al., 2010, 2012). The three FLASH sequences were, respectively, proton density (PD), magnetization transfer (MT), and T1-weighted by choosing appropriate values of TR and flip angle (α) for each of them. The TRs and flip angles for the three FLASH sequences were as follows: PD, TR = 23.7 ms, α = 6°; MT: TR = 23.7 ms, α = 6°; T1: TR = 18.7 ms, α = 20°. For the MT-weighted acquisition, a Gaussian RF pulse of 4 ms duration and 220° nominal flip angle was applied 2 kHz off-resonance before nonselective excitation. Gradient echoes were acquired with alternating readout gradient polarity at 6 equidistant times between 2.2 and 14.7 ms. For PD-weighted acquisition, two additional echoes at 17.2 and 19.7 ms were acquired. A high readout bandwidth of 425 Hz/pixel was used to reduce off-resonance artifacts. During acquisition, subjects were encouraged to be as still as possible with eyes open or closed.

#### Structural data acquisition for use with sound-evoked fMRI

The procedure and parameters for the structural MRI in the sound-evoked fMRI experiment were the same as for the rsfMRI experiment.

#### Analysis

##### Resting state functional connectivity

Functional connectivity analysis was performed using Conn toolbox (version 19.b) (Whitfield-Gabrieli and Nieto-Castanon, 2012). The first five volumes were discarded from the analysis to allow magnetic strength of the scanner to reach steady state. The data were preprocessed using the default preprocessing pipeline (realignment, slice time correction, outlier detection, segmentation and normalization, and smoothing with an 8 mm kernel). A T1-weighted scan acquired as a part of the multiparameter map protocol was used as a structural scan for each subject. The ROIs for auditory cortex (Heschl's gyrus [HG], planum temporale [PT]) were anatomically defined using the brain atlas accompanying Conn toolbox. The ROIs for visual cortex (primary visual cortex V1, secondary visual cortex V2) were extracted from Anatomy toolbox (Eickhoff et al., 2005). The ROIs for dorsal (dPMC) and ventral (vPMC) premotor areas were based on the Human Motor Area Template developed by Mayka et al. (2006). The anterior insula seed regions were based on a sphere of 6 mm radius around the maxima of activation observed in Kumar et al. (2017). The time series was extracted from each voxel of the ROI and then averaged across voxels to define a single time series for the ROI. The effect of movement on the BOLD signal was reduced by regressing out motion parameters, along with their first-order temporal derivative, by running whole-brain voxelwise regression. The effect of physiological noise (cardiac and respiratory) was removed by generating 14 regressors (6 for cardiac phase, 6 for respiratory phase, 1 for heart rate, 1 for change in respiratory volume) using the Physio Toolbox (Hutton et al., 2011) and their first-order temporal derivative and using these regressors of no interest at the first level of analysis. Additionally, five covariates were generated using the aCompCor method (Behzadi et al., 2007), which uses principal component analysis on the measurements made in the white matter and CSF of each individual subject's segmented white matter and CSF masks. The data were bandpass filtered in the range 0.008-0.09 Hz, and first-level functional connectivity for each group was computed using bivariate correlation coefficient between the seed time series and time series from all other voxels in the brain (seed-to-voxel analysis). Comparison of connectivity between the two groups at the second level was undertaken using two-sample *t* tests with “age” and “sex” as regressors of no interest. All results shown are cluster-corrected with (one-sided) cluster defining threshold of *p* = 0.001.

##### Sound-evoked fMRI activations

The analysis was performed using a standard GLM as implemented in SPM12 toolbox (https://www.fil.ion.ucl.ac.uk/spm/software/spm12/). The three categories of sounds were separately modeled as events of 15 s duration and then convolved with the HRF to generate three regressors. Button presses and motion regressors were used as regressors of no interest. Further details of the analysis can be found in Kumar et al. (2017). Interaction between group and sound type in the orofacial motor area was computed using a 2 × 3 ANOVA with group (Misophonia and Control) and sound type (trigger, unpleasant, and neutral) as factors, and subject effects modeled. The ROI for orofacial motor cortex was defined based on the resting state connectivity. Specifically, the part of vPMC which showed stronger connectivity to PT in resting state was chosen as an ROI. Beta values from within an ROI were extracted using in-house MATLAB scripts. Variation of β values in the orofacial motor cortex with rating was estimated using regression analysis and then comparing the regression coefficients between two groups using two-sample *t* test.

##### Sound-evoked functional connectivity

The ROI for the orofacial motor area was chosen as above. Three regressors for the sound categories were defined as in the GLM analysis. Other regressors (cardiac, breathing, and motion) and procedure for analysis were similar to the one used for resting state functional connectivity, except that the data were high pass filtered with a cutoff frequency of 1/125 Hz. All results shown are cluster-corrected with (one-sided) cluster defining the threshold of *p* = 0.001.

## Results

One misophonia subject was excluded from the resting state analysis because the physiological measurements could not be accessed. Three control subjects were excluded because of excessive movements in the MRI scanner. The final sample for analysis of resting state data consisted of 16 misophonia and 17 control subjects.

### Demographics and questionnaire scores

There was no significant difference between age (*p* = 0.38) and sex (*p* = 0.86) of the two groups. The misophonia group scored higher (*p* < 0.001) on the Misophonia Questionnaire (Wu et al., 2014) compared with controls. Misophonia subjects scored an average of 42.6 on the Misophonia Questionnaire compared with 17.6 in controls. Additionally, misophonia subjects scored an average of 15.5 of a maximum of 24 on the Amsterdam Misophonia Questionnaire (Schroder et al., 2013), which corresponds to “severe” misophonia. The demography and questionnaire scores are summarized in Table 2.

**Table 2.** Demographics and questionnaire scores (mean ± SD) for the two groups

	Misophonia	Control
Number of subjects (*N*)	16	17
Sex (female)	8	8
Age (mean ± SD)	38.7 ± 10.3	35.6 ± 9.6
Misophonia Questionnaire (Wu et al., 2014) (symptoms + behavioral)	42.6 ± 10.7	17.6 ± 8.8
Amsterdam Questionnaire (mean ± SD) (Schroder et al., 2013)	15.5 ± 3.4	—

### Resting state functional connectivity

**Figure 1.**
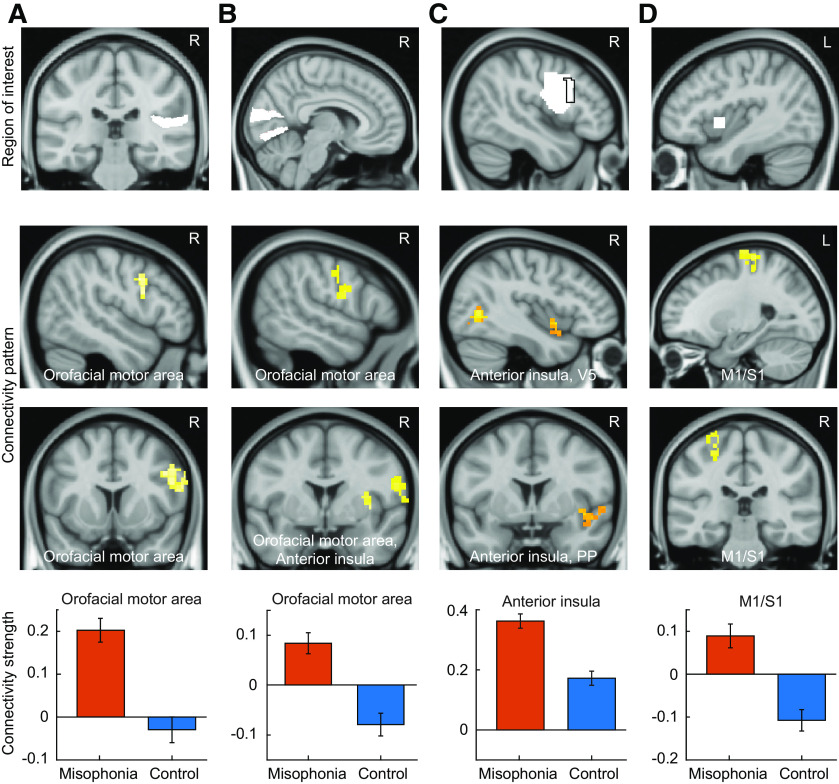
Resting state functional connectivity of (***A***) right PT, (***B***) right V2, (***C***) right vPMC (the black curve in the top panel indicates the boundary of part of vPMC, which shows stronger connectivity to PT), and (***D***) left anterior insula. Connectivity of each of these seed regions was analyzed with the rest of the brain (seed-to-voxel analysis). The results shown are cluster-corrected with a cluster defining threshold of *p* = 0.001. The top row shows the seed regions; the middle two rows show the connectivity pattern overlaid on the sagittal and coronal sections of the structural image; the bottom row shows bar plots of connectivity strength in the two groups. Data in the bar plots represent mean ± SEM.

Since the misophonic reaction begins with audio/visual input, connectivity of auditory and visual cortex was estimated and compared between the two groups. Anatomically defined HG (which contains primary auditory cortex) and PT (secondary auditory cortex) were separately chosen as seed regions, and their connectivity to every other voxel in the brain (seed-to-voxel analysis) was estimated. Relative to controls, the misophonia group showed a significant increase in functional connectivity of right PT to vPMC (Fig. 1*A*; peak at: 60, 12, 24; *t*_(29)_ = 4.62; *p* < 0.001, q(FDR) < 0.05; cluster size = 283). No other cluster in the brain showed significant increased connectivity to PT. There was no significant difference in HG connectivity between the two groups. As in the case of auditory cortex, we chose both primary (V1) and secondary (V2) visual cortex as seed regions. Relative to controls, the misophonia group showed significantly increased connectivity of right V2 to vPMC (Fig. 1*B*; peak at: 58, 0, 24; *t*_(29)_ = 4.34; *p* < 0.001, q(FDR) < 0.05; cluster size = 221; Table 3), which is very close to the part of vPMC that showed increased connectivity to right PT. This area of the motor cortex, when electrically stimulated, produces mouth and lip movements (Kern et al., 2019) and is active when either mouth or lip movements are made (Ehrsson et al., 2003; Grabski et al., 2012; Kern et al., 2019) or when these actions (e.g., someone biting an apple) made by others are passively observed (Buccino et al., 2001; Kern et al., 2019). Moreover, the vPMC is a part of the human mirror neuron system (Rizzolatti and Craighero, 2004). Other areas to which connectivity of V2 was stronger in misophonia included right anterior insula and right parietal operculum/PT (Table 3). There was no significant difference between the connectivity of V1 to vPMC in the two groups, but stronger connectivity of left V1 to right anterior insula and planum polare (Table 3) in the misophonia group was observed.

**Table 3.** List of brain areas that show significant change in resting state connectivity in misophonia compared with controls

Region name	MNI coordinates (mm) of the maxima	No. of voxels	*t* value at the maxima
Stronger connectivity to right V2 in misophonia compared with controls			
Right vPMC	58, 0, 24	221	4.34
Right anterior insula	34, 2,10	180	4.88
Right parietal operculum/PT	30, −34, 20	136	4.94
Stronger connectivity to left V1 in misophonia compared with controls			
Right anterior insula	42, 8, −10	169	4.51
Left planum polare/STG	−60, 6, −4	118	4.84
Lateral occipital cortex, occipital pole	22, −84, 14	978	5.57
Stronger connectivity to right vPMC in misophonia compared with controls			
Occipital cortex, middle temporal gyrus	40, −64, 2	342	5.78
Brainstem	−2, −10, −34	230	6.91
Fusiform gyrus	24, −72, −12	204	5.03
Anterior insula, planum polare, STG	42, 6, −6	165	4.64
Stronger connectivity to the left anterior insula in misophonia compared with controls			
Motor/somatosensory cortex (M1/S1)	−18, −16, 78	383	4.82
Cerebellum (lobule 6)	28, −54, −22	150	5.24

To investigate whether “mirroring” could underlie the misophonic distress, we used rsfMRI data to estimate the connectivity of vPMC, which contains orofacial motor cortex that showed stronger connectivity to PT (Fig. 1*C*, top, boundary marked in black) and the part of anterior insula whose responses most strongly correlated with misophonic distress in our previous work (Kumar et al., 2017). To further test the specificity of connectivity of vPMC, we also chose dPMC as an ROI and estimated its connectivity. Increased connectivity of the right vPMC to anterior insula was observed in the misophonia group (Fig. 1*C*; peak at: 42, 6, −6, *t*_(29)_ = 4.64; *p* < 0.001, q(FDR) < 0.05; cluster size = 165). Other regions included occipital cortex, fusiform gyrus, and middle temporal gyrus (Table 3). No significant difference in the connectivity of left or right dPMC or left vPMC between the two groups was observed indicating the specificity of hyperconnectivity of right vPMC to insula in misophonia.

Finally, the misophonia group also showed a significant increase in connectivity between left anterior insula and left motor and somatosensory (M1/S1) cortex (Fig. 1*D*; peak at: −18, −16, 78; *t*_(29)_ = 4.82; *p* < 0.001, q(FDR) < 0.05; cluster size = 383) and cerebellum (Table 3). This part of the cerebellum (lobule 6) is known to be involved in mirroring and observation of bodily actions of others (Van Overwalle et al., 2014; Abdelgabar et al., 2019). Stronger coupling of vPMC containing orofacial motor cortex and cerebellum to anterior insula supports “mirroring” as a likely mechanism for the autonomic and emotional reaction in misophonia mediated by the anterior insula.

### Functional connectivity of orofacial motor area in response to sounds

**Figure 2.**
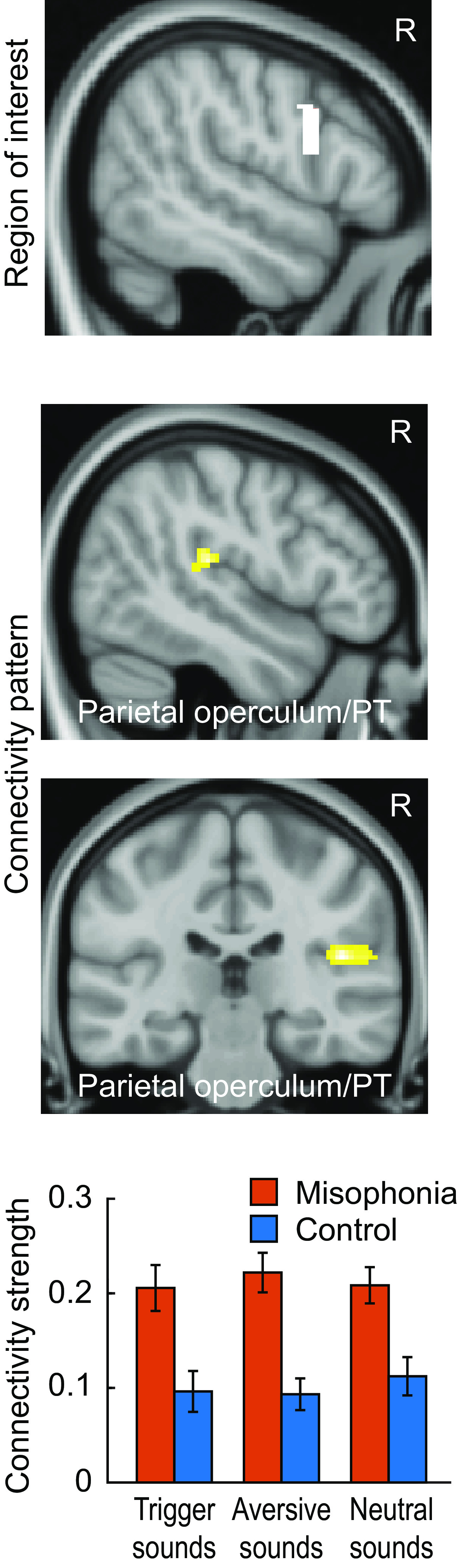
Sound-evoked functional connectivity of right orofacial motor cortex. The orofacial motor ROI (top) is selected from the resting state connectivity analysis. The two middle rows show brain regions with increased connectivity in the misophonia group in response to all sounds. Bar plot in the bottom row plots connectivity strengths in the two groups in response to sounds. The results shown are cluster-corrected with a cluster defining threshold of *p* = 0.001. Data in the bar plots represent mean ± SEM.

We next examined sound-driven changes in functional connectivity. This was done using fMRI data acquired on a separate misophonia and control cohort during the presentation with three categories of sounds: trigger sounds (which evoke misophonic reaction in the misophonia group), unpleasant sounds (which are perceived as aversive by both the groups but do not evoke misophonic reaction in the misophonia group), and neutral sounds (for rating data, see Kumar et al., 2017, their Fig. 1). We first defined an ROI in the orofacial area showing stronger connectivity to PT in the resting state and then analyzed its connectivity to the rest of the brain in response to sounds. A significant main effect of group (Misophonia Group > Controls) was observed in the auditory cortex covering right PT and right lateral HG (Fig. 2, top; peak at: 46, −26, 16; *p* < 0.001, q(FDR) < 0.05; cluster size = 137), but no significant group × sound interaction was seen in the auditory cortex. Plots of connectivity values (Fig. 2, bottom) in the auditory cortex confirm the main effect.

### Activation of the orofacial motor area to sounds

**Figure 3.**
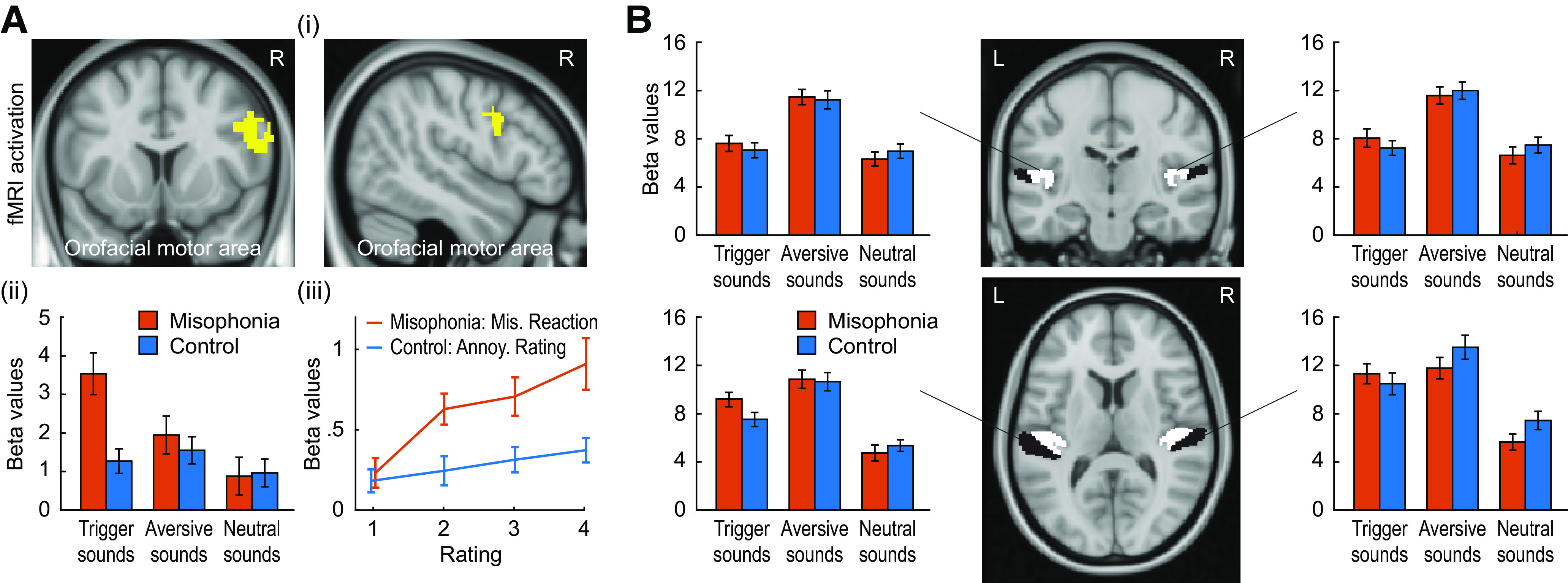
Activation of orofacial motor cortex and auditory cortex in response to three categories of sounds. ***Ai***, The orofacial motor area represents a statistically significant (*p* = 0.002) group × sound category interaction. ***Aii***, Bar plots represent β values for the orofacial motor cortex for the two groups in response to three sound categories. ***Aiii***, Plots of variation of β values with the rating of misophonic distress in misophonia sufferers and of annoyance in control subjects. No group × sound category interaction is seen in the auditory cortex (***B***, middle column), which is confirmed by the bar plots of activation (***B***, first and third column) in response to sounds for the two groups. ***B***, Middle column: white represents HG; black represents PT. Data in the bar plots represent mean ± SEM.

The increased connectivity between audiovisual sensory and orofacial motor cortical areas in misophonia in the resting state and in the presence of auditory stimulation made us consider whether the orofacial motor area would show increased activation to trigger sounds in the misophonia group. With the ROI of the orofacial motor area as defined above, a significant group × sound category interaction was observed (Fig. 3*Ai*; *p* = 0.002 after small volume correction). Plot of β values (Fig. 3*Aii*) for individual sound categories shows that the interaction is driven by greater activity in misophonia subjects compared with controls for trigger sounds but not for unpleasant or neutral sounds. Importantly, this specific increase in activity for trigger sounds was not shown in auditory cortex (Fig. 3*B*), suggesting that a trigger sounds-specific increase in activity first arises in the orofacial motor cortex, rather than being carried forward from earlier auditory system hyperactivity.

To further confirm the relation between behavioral data and BOLD activity in the orofacial motor cortex, we measured activity (β values) for each sound individually (without reference to the group to which it belongs) and determined how the activity of orofacial motor cortex relates to the rating given to the sound by the subjects. Figure 3*Aiii* plots the variation of β values with misophonic distress rating for misophonia sufferers and annoyance ratings for control subjects. As can be seen, the activity of orofacial motor area increases in proportion to the rating. However, this increase is stronger (*t*_(40)_ = 6.8, *p* < 0.001) for misophonia sufferers compared with controls.

## Discussion

In this study, we have demonstrated that misophonia is characterized by (1) increased resting state functional connectivity between the orofacial motor area and both auditory and visual cortex; (2) increased functional connectivity between auditory cortex and orofacial motor areas in response to all types of sound; (3) increased activation of orofacial motor area in response to trigger sounds specifically; (4) activation of orofacial motor area increases in proportion to the misophonic distress; (5) no difference from the control group in the activation of auditory cortex to trigger and other sounds; and (6) increased resting state functional connectivity between vPMC containing orofacial motor area and insula in the resting state.

Conventionally, misophonia is considered as a disorder of sound emotion processing, in which “simple” sounds of eating/chewing produced by others cause abnormally high negative emotional responses. However, sounds generated by others can represent the actions that produce them (Aglioti and Pazzaglia, 2010) and can also modulate the actions of listeners (Ferrari et al., 2005; Aglioti and Pazzaglia, 2010). We consider here whether sounds in misophonia may be only a “medium” via which action of the trigger-person is mirrored onto the listeners. In that case, the primary abnormality might be excessive engagement by the auditory system, and/or intrinsic hyper-responsiveness of, the orofacial motor system. The present findings of normal auditory cortex responses to trigger sounds, yet increased connectivity between auditory and orofacial motor areas and orofacial motor hyper-responsiveness specifically to trigger sounds, support this notion.

Resting state connectivity measures spontaneous fluctuations of brain activity, which are correlated between two regions. It is known to be predictive of task/stimulus activations of brain regions (Tavor et al., 2016; Parker Jones et al., 2017) and behavior (Spisak et al., 2020). Our results show that spontaneous fluctuations in auditory/visual cortex and orofacial motor cortex are synchronized to a greater extent in a misophonia group compared with the control group. This stronger spontaneous coupling between auditory or visual and orofacial motor cortex in the absence of any systematic external stimulation implies that the orofacial motor cortex in misophonia sufferers is primed to respond to sensory stimulation related to the production of trigger sounds.

Further evidence that input sounds in misophonia are transformed into motor representations comes from stronger coupling of orofacial motor areas to auditory cortex in response to auditory stimulation. However, this increased connectivity is in response to sounds in general and not specific to trigger sounds. Increased resting state connectivity between the auditory and motor cortex in misophonia is in the absence of any sound stimulation and, therefore, also not specific to trigger sounds. Since functional connectivity, particularly in the resting state, is known to be constrained and explained by structural connectivity (Greicius et al., 2009; Honey et al., 2009), it is likely that in misophonia there is a stronger structural connectivity between the auditory and orofacial motor cortices. This needs to be investigated in future studies.

Does stronger coupling to auditory cortex in the resting state translate to stronger activation of orofacial motor cortex? Our findings suggest that, in misophonia, trigger sounds cause hyperactivation of the orofacial motor cortex implying possible excessive “mirroring” of the orofacial actions via trigger sounds. Although this mirroring of action via sensory stimuli occurs in normal and healthy subjects (Buccino et al., 2001), the mirroring is stronger (hyper-mirroring) and specific to trigger sounds in misophonia. Importantly, such specificity of hyperactivation to trigger sounds is absent in the auditory cortex, which further demonstrates that misophonia is a not a disorder of sound processing per se but relates to the (orofacial) actions that the sounds represent. Putting together results of resting state connectivity, sound-evoked functional connectivity, and activation, our data strongly support the hypothesis that misophonia is characterized by aberrant functioning of the mirror neuron system which “mirrors” the action of the trigger-person represented by sounds.

A consequence of mirroring of action is that it leads to “automatic imitation” (Iacoboni et al., 1999; Heyes, 2011) in which subjects mimic the action of the producer. This imitation, which is unconscious and unintentional, facilitates actions and responses that are congruent with the actions of the producer (e.g., mouth movement in response to eating sounds of the trigger-person). What exactly makes excessive mirroring trigger the extreme negative characteristic reaction in misophonia is presently uncertain, but possibilities include a sense of loss of control, invasion of personal space, or interference with current goals and actions. A recent study, using a Stroop task, did show that misophonia sufferers are impaired in maintaining goals of the task, specifically in the presence of trigger sounds (Daniels et al., 2020). Since anger can be described as perceived goal interference (Hufendiek, 2016), the drive to imitate others would make anger (or aggression) the dominating emotional response. This is consistent with the phenomenological reports of emotional responses to trigger sounds in misophonia (Jager et al., 2020). Mimicry of the action producing the trigger sound may be a direct consequence of mirror system activation (i.e., overt as opposed to covert), or a coping strategy (Edelstein et al., 2013; Taylor, 2017) to dampen sensory activity, much like attenuation of sensory input following a self-generated action (Blakemore et al., 2000).

Interestingly, outside of the context of misophonia, automatic mimicry of eating actions is common among family members (Hermans et al., 2012; Bell et al., 2019), which may explain why a family member most commonly acts as a source of triggers in the initial phase of misophonia. Although we have confined our discussions to sounds of eating/chewing as trigger, our model of hyper-mirroring in misophonia can be used to explain less-common visual triggers, such as foot/leg shaking because mimicking of these actions occurs in normal subjects (Chartrand and Bargh, 1999).

Our previous work (Kumar et al., 2017) showed that misophonic distress correlates with the activity of anterior insula. The current results on resting state connectivity indicate stronger connectivity of orofacial motor cortex to insular cortex in misophonia, providing a link between mirroring of action, insular activity, and misophonic distress. Moreover, anterior insula has stronger resting state connectivity to cerebellum (lobule 6), which is known to be involved in social cognition in general and mirroring of actions of others in particular (Van Overwalle et al., 2014). Interestingly, neuroimaging of automatic imitation in normal subjects shows that countering of mirrored actions (e.g., closing one's hand when viewing a hand opening and vice versa), either intentionally or incidentally, engages anterior insula (Campbell et al., 2018). In summary, our data support that mirroring of action underlies the previously observed anterior insula-based network in misophonia.

Misophonia as an aversive “reflex” has been argued previously by Dozier (2015). In this model, sound triggers a reflex-like bodily (physical) response, which is then followed by emotional response. While our data are in agreement with the larger point of the model emphasizing role of motor system in misophonia, the fact that higher-order motor cortex is involved suggests a complex role of the motor system not consistent with the reflex-like response in the Dozier (2015) model. It is, however, likely that the mirroring response is automatically triggered from the vPMC and subsequently expands to other part of motor cortex, including primary motor cortex, which can explain sensations and muscle activity in other parts of the body in response to trigger sounds.

Our data provide an alternative but complementary perspective on misophonia that emphasizes the action of the trigger-person rather than the sounds which are a byproduct of that action. In doing so, misophonia can be understood within a wider framework of social cognition, which concerns how people perceive auditory/visual cues from other people. This change in perspective has important consequences for devising therapies and treatment methods for misophonia. For instance, associative learning has been shown to configure the MNS (Catmur et al., 2007): if this process could be harnessed to associate misophonic trigger sounds with sound sources other than orofacial actions, then they might no longer evoke the misophonic reaction. Evidence for association having effects on perceived aversiveness of sounds comes from Samermit et al. (2019) in which aversive sounds were either associated with a positive or negative source. The same sound when associated with a positive source was rated as less unpleasant and also produced fewer bodily sensations compared with when association was with its original negative source. How this association is mediated by the MNS needs to be investigated further.

Finally, we outline some limitations of our work and the future direction to overcome these limitations. First, demonstration of mirror neurons requires invasive single-neuron recording, which is not normally possible in humans. Measurements using fMRI are too coarse at spatial and temporal resolution to reveal the working of single neurons. Our evidence of involvement of mirror neurons in misophonia, therefore, like most of the human neuroimaging studies implicating mirror neurons, is indirect. There have been a few studies of single-cell recording demonstrating mirror neurons in human epileptic patients undergoing neurosurgery (Mukamel et al., 2010). Future studies may use such rare opportunities to provide direct evidence for the role of mirror neurons in misophonia. Second, although the distinctness of misophonia as a disorder on its own has been argued (Schroder et al., 2013; Erfanian et al., 2019; Swedo et al., 2021), there is still debate (Taylor, 2017) of how much of it can be explained by other disorders. In order to test its distinctness or overlap with respect to functioning of the mirror neuron system, future studies could include a clinical control group in addition to the normal healthy control group. Third, although less common compared with typical triggers, some misophonia sufferers also report nonhuman (e.g., animal sounds) and environmental sounds as their triggers (Jager et al., 2020). How does our hyper-mirroring model of misophonia explain distress to these triggers? It should be noted that nonhuman and environmental sounds as triggers in misophonia do not occur in isolation. That is, these nontypical triggers occur along with the typical triggers (e.g., eating/chewing sounds) in misophonia sufferers (Muller et al., 2018; Jager et al., 2020; Wiese et al., 2021). A plausible explanation, therefore, is that, after the typical triggers are learned via the mirror system, the nontypical stimuli become triggers via associative learning. This is also consistent with the observation that the set of trigger stimuli for misophonia sufferers expand over time. Further work, however, is needed to empirically validate this hypothesis.
